# Point‐of‐care ultrasound: Closing guideline gaps in screening for valvular heart disease

**DOI:** 10.1002/clc.23499

**Published:** 2020-11-11

**Authors:** Muhammad Hammadah, Christopher Ponce, Paul Sorajja, João L. Cavalcante, Santiago Garcia, Mario Gössl

**Affiliations:** ^1^ Interventional Cardiology Department Minneapolis Heart institute Minneapolis Minnesota USA

**Keywords:** POCUS, point of care ultrasound, screening, valvular heart disease

## Abstract

**Background:**

A linear increase in the number of valvular heart disease is expected due to the aging population, yet most patients with severe valvular heart disease remain undiagnosed.

**Hypothesis:**

POCUS can serve as a screening tool for valvular heart disease.

**Methods:**

We reviewed the literature to assess the strengths and limitations of POCUS in screening and diagnosing valvular heart disease.

**Results:**

POCUS is an accurate, affordable, accessible, and comprehensive tool. It has a fast learning curve and can prevent unnecessary and more expensive imaging. Challenges include training availability, lack of simplified screening protocols, and reimbursement. Large scale valvular screening data utilizing POCUS is not available.

**Conclusion:**

POCUS can serve as a screening tool and guide the management of patients with valvular heart disease. More data is needed about its efficacy and cost‐effectiveness in the screening of patients with valvular heart disease.

## 1 INTRODUCTION

As the population continues to age, the adverse effects associated with valvular heart disease are increasing as well.^1^ With the introduction of the transcatheter aortic valve replacement (TAVR), the number of patients who will be eligible for TAVR is estimated to increase 4‐fold over the next 5 years.^2^ Similarly, the number of patients who may benefit from transcatheter mitral valvular interventions such as Mitraclip is expected to increase.^3^ Unfortunately, many patients with severe valvular heart disease remain undiagnosed.^4^, ^5^ One possible limiting factor is relying only on physical exam as an initial screening test for detecting valvular abnormalities. One study that surveyed a group of primary care physicians and cardiologist showed that the confidence of the physicians to detect mitral regurgitation with a stethoscope was less than 50%.^5^, ^6^ Thus, to meet such demand, a reliable and accessible technology is essential to enhance the physical exam quality and sensitivity in screening for valvular heart disease. The prevalence of moderate or severe valvular heart disease in a large‐scale community screening program of patients over 65 years in the United Kingdom exceeded 11%, with a projected doubling before 2050.^4^ An early detection of valvular heart disease, through echocardiograms, can have a lasting impact on morbidity, mortality, and cost of care.^7^

With the advancement in technology, portable ultrasound machines became readily available. These include both stationary high‐end ultrasound system, and small handheld ultrasound devices (HUD). Point of care ultrasound (POCUS) refers to a goal‐oriented, limited ultrasound examination of a particular body structure with a predefined limited protocol.^8^ Performing POCUS using HUD is an accurate, affordable, accessible tool that can aid physical exam and streamline unnecessary clinical testing.^6^, ^9^ In addition, visual representation allows physicians to glimpse inside the patient to better examine and diagnose a condition.^6^, ^9^ POCUS has a high correlation with standard echocardiogram in evaluating left ventricular function and valvular abnormalities, making it a potential useful tool for screening for valvular abnormalities. We aim in this article to review the role of POCUS in cardiac evaluation and its potential rule in screening for valvular heart disease.

## 2 CURRENT SCREENING METHODS FOR VALVULAR HEART DISEASE ‐ A ROOM FOR IMPROVEMENT

The current screening practice of valvular heart disease is mainly dependent on cardiac auscultation. Only those with abnormal findings, are referred for a standard echocardiogram. There are no clear recommendations about who and how to screen by neither the American college of cardiology, nor American heart association.^7^ The stethoscope, a 200 year old device discovered by Dr Laennec, remains the solo screening tool for valvular abnormalities.^10^, ^11^ Yet the amount of information that can be gained through auscultation is not comparable to that of the ultrasound.^12^ Expert cardiologists still experience the limitation of auscultation when it comes to confidently diagnosing valvular abnormalities.^11^, ^13^, ^14^, ^15^ POCUS has been shown to perform better than traditional auscultation methods in evaluating valvular heart disease, even when carried‐out by non‐cardiologists.^13^, ^16^, ^17^, ^18^, ^19^ Initial diagnosis gathered through traditional methods like auscultation, can be easily verified using POCUS, making ultrasound imaging a better option.^20^ Therefore, performing POCUS examination improves the accuracy of diagnosis of valvular heart disease from 50% to 80% in as little as 15 minutes after a patient exam has started.^21^ After the introduction of HUD, physicians have been able to increase the range of acute and chronic conditions that can be diagnosed using POCUS.^22^ One small study has compared the performance of board certified cardiologists utilizing standard physical exams, with medical students trained for 18 hours to perform POCUS using HUD. The use of POCUS outperformed the experienced cardiologists in detecting abnormal cardiac pathologies (75% accuracy), and valvular pathology (93% vs 49%).^23^ Another study has compared the results of physical examination performed by board certified cardiologist with the results of POCUS in a sample of 36 patients with cardiovascular disease. Cardiac examination alone failed to detect 59% of the overall cardiovascular findings and missed about 43% of the major findings. POCUS reduced this to 21% without significant inter‐physician variation.^13^ Thus, POCUS can reduce the time it takes to reach a conclusion and the price of the device can reduce the cost of echocardiography as well.^6^, ^24^, ^25^, ^26^, ^27^

## 3 HUD VS STANDARD ECHOCARDIOGRAPHY

Though portable echocardiogram devices have the potential to enhance auscultation, they are by no means a substitute for standard echocardiography. Therefore, its optimal role in healthcare has yet to be officially defined. Currently, standard echocardiography machines tend to be too big and expensive for primary care medical clinics, potentially hindering immediate ultrasound access.^9^, ^13^ Performing POCUS using HUD has been shown by one study to decrease the number of rarely appropriate standard echocardiography by 59%.^6^, ^9^ The study by Vourvouri et al, has shown that screening with point of care ultrasound avoids the use of standard echocardiography in approximately 80% of unselected patients and leads to a 33% cost reduction.^28^

HUDs are equipped with color Doppler, which can provide a qualitative evaluation of valvular heart disease. However, the lack of spectral Doppler limits their ability for quantitative assessment.^6^, ^24^, ^25^, ^26^, ^29^, ^30^ HUDs have good sensitivity and specificity in evaluating regional wall motion abnormality and left ventricular global function. However, they have only modest accuracy in evaluating valvular heart abnormalities such as severe aortic stenosis, and mitral/tricuspid valve regurgitation.^6^, ^8^, ^24^, ^25^, ^26^, ^31^, ^32^ POCUS using HUD has been shown to have a very good sensitivity and specificity for diagnosis of rheumatic heart disease.^31^, ^33^ Several devices are now equipped with online internet connection and Cloud image storage. Newer devices are also equipped with artificial intelligence which can guide the provider how to improve image acquisition.^34^

## 4 HUD AND POCUS IN CLINICAL PRACTICE

With the expansion of availability of HUD and portable ultrasound stations, there have been several developments on how they can be utilized.^35^ POCUS was first utilized in trauma patients in the 1970s in Europe, and later on was adopted in the US in the 1990s, mainly in the emergency department where the health care provider can rapidly identify life threatening conditions, such as pericardial effusion, pneumothorax, and bowel injury.^33^, ^36^, ^37^ Several protocols were adopted by different centers and utilized by providers with variable degree of training.^20^, ^26^, ^38^, ^39^, ^40^, ^41^, ^42^, ^43^, ^44^, ^45^ Currently, POCUS is mainly used in the emergency department and intensive care units. Its role in non‐emergent settings is still emerging. Among non‐emergent conditions, POCUS can improve physical exam,^46^ guide diuresis,^47^ predict risk of rehospitalization,^48^ and safely discharge cardiac patients from the clinic.^36^

## 5 POCUS TRAINING AND CHALLENGES

Although the HUD has been available since the 1970s, there is still hesitancy by many physicians about incorporating POCUS in their clinical practice, mainly due to a lack of formal rules or guidance on when and how a proper exam should be conducted.^21^, ^26^, ^35^, ^49^, ^50^ Given its importance, increasing availability and high accuracy, POCUS could became an essential part of the physical exam and can provide comparable information to auscultation and palpation.^26^, ^50^ Limitations in training availability has slowed its implementation. Though progress has been slow, there has been an increase in POCUS training in undergraduate and graduate level and continuing in professional programs.^51^, ^52^ Recently, Harvard medical school incorporated POCUS training in first‐ and second‐year medical students' curriculum as part of the physical exam training.^53^, ^54^ Similarly, residency programs have included POCUS training in their curriculum.^54^, ^55^ Though some guidelines currently exist, there is a need for new and updated data on the current methods of practicing.^5^

The American Society of Echocardiography has predicted an exponentially increasing number of POCUS users and has emphasized the importance of high quality training, interpretation, and proper usage.^29^ Several efforts are being made to encourage physicians to use ultrasound in their practice with hopes of reducing the healthcare cost by not requesting more expensive imaging.^22^ It became crucial to better understand the possibilities and necessities for POCUS, and to have guidelines and protocols for its use. For example, the focused assessment with sonography in trauma (FAST) protocol has been widely adopted due to its ease and accuracy, and importantly speeding up and advancing patient care.^39^, ^42^, ^56^, ^57^ Currently, the only field that has a dedicated and clear echocardiography training is cardiology. Cardiology fellows need to perform and read certain numbers of echocardiograms, as assigned by the American college of cardiology, to achieve different levels of competencies.^58^ The American Society of echocardiography and American college of emergency physician have issued consensus statement about the use of focused cardiac ultrasound in emergency settings.^57^ The American college of chest physician (ACCP) as well as several critical care societies have issued several requirements on competencies in critical care ultrasonography and echocardiography.^59^, ^60^, ^61^, ^62^ Unfortunately, POCUS training is still not a requirement nor formally incorporated during internal medicine training.^63^ A 2014 survey of family medicine program directors found that only 2% of residency programs had a formal POCUS curriculum.^64^ There is still no consensus on the training requirements to achieve adequate competency level. However, it's generally agreed that training must include basic ultrasound physics knowledge, supervised image acquisition and interpretation.^46^ Some studies showed residents could perform accurate echocardiograms after a training for several hours to only a few days,^54^, ^65^, ^66^ or as little as 25 scanning exams.^60^

## 6 POCUS IN PRIMARY CARE SETTING

**FIGURE 1:**
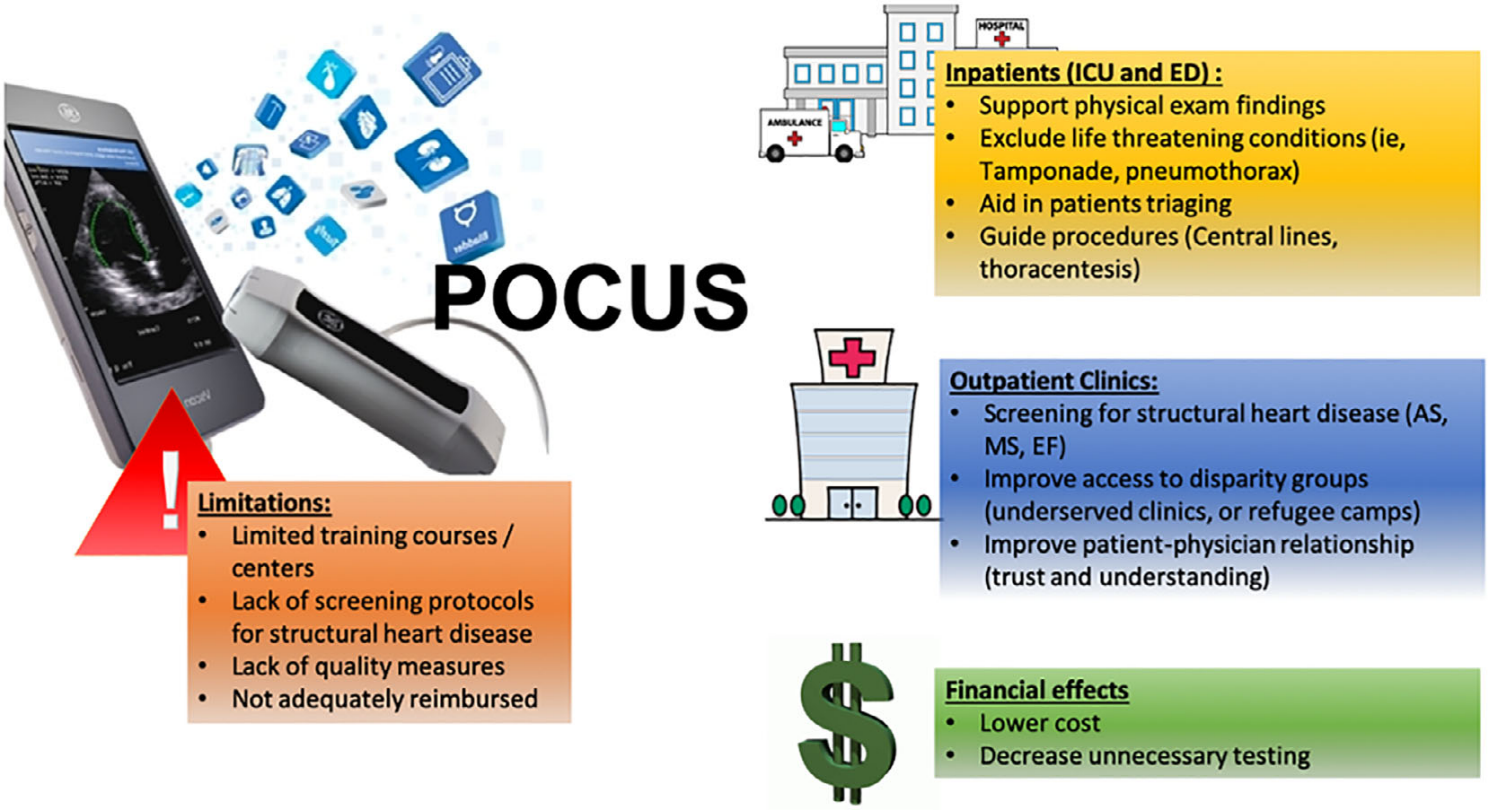
Clinical application and limitations of point of care ultrasound

In the outpatient setting, the utilization of HUD is also gradually increasing.^64^ This technological innovation has brought the clinician closer to patients when it comes to diagnosis, increasing the relationship and overall patient satisfaction.^67^, ^68^ POCUS allows patients and physicians to view images together where changes can be easily seen and tracked, and images can serve as a guide to explain more physiological concepts.^69^ (Figure 1) This can also improve the quality of care and patient safety.^69^ With the advancement of transcatheter treatment, screening for valvular heart disease in the outpatient setting might help in early detection and treatment, which may prevent downstream complications of valvular heart disease.^4^, ^7^, ^68^, ^70^ In a recent randomized clinical trial that assessed patient with known structural heart disease, the addition of POCUS to clinic evaluation resulted in earlier referral for valvular intervention, and decreased the risk of hospitalization and mortality.^27^ Given its ease and low cost, POCUS using HUD will be the tool of choice to screen high risk patients.^4^ Patients with any valvular abnormalities, can be confirmed by standard echocardiography. Those with valvular abnormalities, can also be followed using POCUS without the need for a more expensive echocardiogram.^27^ There is an increasing evidence, mainly from low to middle income countries, that HUD can be used to screen for structural heart disease and can improve patients outcomes.^27^, ^31^, ^33^, ^71^, ^72^, ^73^, ^74^, ^75^

In comparison to the in hospital setting where POCUS can be easily performed in the emergency department and critical care units, POCUS availability and utilization in the primary care setting is still limited and faces multiple challenges including device availability, lack of specific screening protocols, training programs and reimbursement.^64^, ^66^, ^67^ With the rapidly growing technology, the cost of HUD is expected to drop significantly, and when compared to the formal echocardiography, they are at least 10 times cheaper.^66^ A simplified protocol for screening for valvular heart disease and cardiac abnormalities is needed before POCUS can be used as a practical tool to screen for valvular heart disease. Although it has not been widely adopted, a brief focused POCUS training focused on valvular heart disease and cardiac function is promising.^55^ One study has implemented a POCUS training program consist of a 50‐question test, 4‐lectures on basic echocardiography & imaging interpretation, a supervised interpretation of 50 echocardiograms, and performance of 30 exams using HUD.^55^ They have trained 12 residents, and compared their performance in 30 cases to experienced cardiologist. The performance of the trained residents was comparable to experienced cardiologist in detecting normal findings (95% correct interpretation), and to a lesser degree of abnormal findings (75% correct interpretation). The performance of the residents was also very good in detecting valve abnormalities (85% correct interpretation).^55^ It remains that, the biggest limitation to this new technology is the fact that there are not enough trainers of POCUS.^55^ Learning programs, like the GE Digital Expert that provides virtual and flexible face to face training could also be tailored to teach physicians POCUS and help face some of these challenges mentioned in this review.^59^

## 7 USING POCUS IN SCREENING FOR VALVULAR HEART DISEASE

The data about using POCUS in screening for valvular heart disease is scarce. Most of the published studies have enrolled small samples of subjects with and without valvular heart disease and focused on sensitivity and accuracy of detecting valvular heart abnormalities. The use POCUS on a large scale to screen individuals without known valvular heart disease yet faces multiple challenges. The prevalence of valvular heart disease increases with age, and this will have implications on the sensitivity and specificity of any screening tests. The prevalence of rheumatic heart disease is higher in younger age and estimated at 12.9 per 1000 people in developing countries,^74^ while its exceedingly rare in the United States (~0.04 cases per 1000 children).^74^, ^75^ In a landmark U.S. study by Nkomo et al, that included 11 911 subjects who prospectively underwent echocardiographic examination in three large national population‐based epidemiological studies: the CARDIA (Coronary Artery Risk Development in Young Adults),^76^ ARIC (Atherosclerosis Risk In Communities),^77^ and CHS (Cardiovascular Health Study),^78^ the age‐adjusted prevalence of moderate or severe valvular heart disease was 2.5%, and was significantly influenced by age: <2.0% prevalence in those <65 years of age and 13.2% in those ≥75 years of age. Increasing age (per 10 years) was significantly associated with mitral regurgitation (odds ratio of 1.84; 95% CI: 1.70 to 1.99; *P* < .0001), mitral stenosis (odds ratio of 1.65; 95% CI: 1.12 to 2.43; *P* = .01), aortic regurgitation (odds ratio of 1.49; 95% CI: 1.30 to 1.70; *P* < .0001), and aortic valve stenosis (odds ratio of 2.51; 95% CI: 2.02 to 3.12; *P* < .0001). In subjects ≥75 years of age, the most frequent valvular heart disease was mitral regurgitation (9.3%), followed by aortic stenosis (2.8%), aortic regurgitation (2.0%) and mitral stenosis (0.2%).^79^ The prevalence of moderate to severe aortic stenosis was also reported to range between 2.9% ‐ 4% in other smaller studies.^80^, ^81^ In a systematic review and meta‐analysis including 9723 patients >75 years of age reported that the prevalence of AS was 12.4%, while severe AS was 3.4%.^82^ Another study has reported the prevalence of bicuspid aortic valve to be 22% among octogenarians patients undergoing aortic valve surgery.^83^ In the Framingham heart study that screened 1696 men and 1893 women (aged 54 +/− 10 years) for valvular regurgitation during routine examination, the prevalence of at least mild mitral regurgitation and tricuspid regurgitation was 19.0% and 14.8% in men, and 19.1% and 18.4% in women, respectively.^84^ In the OxVALVE population cohort study that screened individuals aged≥65 years from a primary care population without known valvular heart disease, the prevalence of any valvular heart disease was 51% of participants.^4^ The most common abnormalities were aortic sclerosis (34%), mitral regurgitation (22%), and aortic regurgitation (15%). The prevalence increased linearly with age, from 42.4% (379/894) in those aged 65 to 69 years to 76.3% (103/135) in those aged 85 to 95 years. The proportion with moderate or severe valvular heart disease was 3.3% (54/1621) among those aged 65 to 74 years, rising to 11.9% (105/879) in those aged ≥75 years. Based on these results, the yield of screening of valvular heart disease might be more effective in the elderly, particularly those older than 75 years.

The other challenge in screening for valvular heart disease is how asymptomatic patients should be treated. The current American college of cardiology / American heart association guidelines largely recommend treatment only in those with severe valvular heart disease who are symptomatic, or with evidence of left ventricular or right ventricular dysfunction.^7^, ^68^, ^70^ Those without are usually recommended to get periodic echocardiograms. It's possible that early diagnosis of valvular dysfunction may lead to additional imaging and additional cost. Ongoing trials such as early TAVR in asymptomatic aortic stenosis patients might help in addressing this question. Indeed, prospective data about large scale screening and its effect on outcomes and health care cost is needed.

The other potential group that may benefit from screening using POCUS are the young athletes before participating in strenuous competitive exercises. Sudden cardiac death remains a devastating event among athletes, and the European society of cardiology recommends history, physical exam, and electrocardiogram screening for these individuals.^85^ Several studies have utilized POCUS in screening athletes for different causes of sudden cardiac death, such as hypertrophic cardiomyopathy, and anomalous coronary artery. Using a simplified protocol to assess septal wall thickness, left ventricular function, and aortic root dilation, POCUS has been shown to be an easy and effective method for screening for hypertrophic cardiomyopathy.^86^ A large metanalysis that included five studies, representing 2646 athletes, demonstrated that electrocardiogram‐inclusive preparticipation screening strategies for potential causes of sudden cardiac death, resulted in positive results in 19.9% of the cohort. With the addition of POCUS, positive results were reduced to 4.9%, and 1 additional condition potentially associated with sudden cardiac death was identified.^87^ The cost of POCUS was reported to range between $20 to $28 per athlete / student screened.^86^, ^88^ If the cost to perform POCUS is modest, and it results in a reduction in false‐positive results and subsequent secondary investigations and cardiologic consultations, then POCUS may represent a cost‐saving screening modality. However, there are insufficient data to draw conclusions regarding cost‐effectiveness from these studies.^87^

In developing countries, POCUS has been utilized by several large‐scale studies to screen for rheumatic heart disease.^27^, ^31^, ^33^, ^71^, ^72^, ^73^, ^89^, ^90^ The world health organization has developed a standard echocardiography protocol for screening, and the widely available handheld devices allowed for active screening and identification of undiagnosed and subclinical carditis in endemic areas. Most of the studies have screened school students.^31^, ^91^ In comparison to a standard echocardiogram, POCUS has been shown to be equally effective in the diagnosis of definite rheumatic heart disease with comparable sensitivity and specificity.^31^ More recent studies focused on training non‐expert healthcare workers (eg, nurses, medical students, clinical officers),^72^, ^92^ in using POCUS to screen for rheumatic heart disease have shown a promise. This might be a viable strategy to implement a screening program in areas where there is a deficiency of highly trained sonographers or cardiologists.^93^

## 8 POCUS CHALLENGES AND LIMITATIONS

With the decreased resources available and increased need for healthcare, new medical innovations are always being evaluated for cost, effectiveness, and reliability.^50^ Individual reimbursement still remains an important barrier for its more ubiquitous use.^29^ This is especially true in clinics where physicians get compensated through the relative value unit (RVU).^29^ In a RVU‐based compensation system, POCUS exam may cause financial loss to the practice, due to increased time of the clinic visit, not appropriate compensation, and less income from ordering additional imaging tests.^6^ POCUS was shown to be very successful in reimbursement systems based on time (not RVUs), where the total cost of care matters more than individual income.^66^ To improve efficiency, some groups have added a *“POCUS clinic”* to their primary care clinic, where patients felt to benefit from imaging can get scanned without interrupting the clinic workflow.^66^ A future shift in reimbursement based on quality of care rather than quantity, may help address this issue. A proper documentation of the indication and findings, as well as image storage are essential for reimbursement. Additional system barriers include availability of training programs, unclear credentialing requirements, efficiency, electronic storage for image archiving, and policies and procedures for quality assurance.^46^

It's important to note that most of the published studies about POCUS utilization are designed to show feasibility and accuracy of POCUS with little or no evidence how it can impact patients' outcomes and healthcare costs. It's possible that POCUS may lead to a higher number of unnecessary testing due to false positive findings. Further studies are needed.^8^

## 9 A PROPOSED TRAINING PROGRAM

As stated earlier in the manuscript, there is no consensus about training protocols and the minimum amount of training needed for cardiac POCUS. It's generally agreed that training must include basic ultrasound physics knowledge, supervised image acquisition, and interpretation.^46^, ^94^ Several protocols were proposed by small studies to train non‐cardiologist or non‐sonographers. The VISION‐in‐Tele‐Echo protocol^95^ was validated in two separate cohorts and could serve as a training protocol for those interested in developing a screening program for structural heart disease.^27^, ^95^ The program was first developed to train 17 non‐cardiologist physicians to performed cardiac POCUS. The protocol consists of 6 hours of focused training in echocardiography in a tertiary care center. The training was performed in‐site for nine physicians, and remotely in the remaining ones. The training was performed by expert sonographers who were American society of echocardiography members. The training began with a 1‐hour lecture that introduced the fundamentals of basic echocardiographic examination and oriented the participants to the specifically designed scanning protocol. The scanning protocol consisted of 11 standard views, including color‐flow Doppler images of all valves. The standard views included: 2D and color images of parasternal long, parasternal short, apical four‐chamber, and apical five‐chamber views. This was followed by hands‐on training using the pocket‐size and HUD units. The trained physicians subsequently scanned elderly patients undergoing cataract surgery. The quality of images was graded, and agreement between local physicians' interpretations and Web‐based interpretations by worldwide experts was compared. A total of 968 studies were performed, 660 were used for validating physicians' competence. The trained physicians could recognize the major echocardiographic abnormalities with 58.7% sensitivity and 97.0% specificity (overall k = 0.62, *P* < .001). Diagnostic accuracy was the best for valve lesions (sensitivity, 80.9%; specificity, 99.8%; k = 0.88; *P* < .001) and relatively modest for left ventricular systolic dysfunction (sensitivity, 58.0%; specificity, 98.3%; k = 0.62; *P* < .001).^95^ This protocol was then utilized in a randomized control trial lead by the American society of echocardiography foundation (ASEF‐VALUES; American Society of Echocardiography Foundation‐Valvular Assessment Leading to Unexplored Echocardiographic Stratagems). POCUS in this study was performed by non‐cardiologist physicians. Among patients with structural heart disease, and in comparison, to standard clinical practice, the utilization of POCUS has led to early referral for valvular interventions and a lower probability of hospitalization or death.^27^ Readers in both of these cohorts were requested to give only visual, and qualitative assessments (mild, moderate, or severe) on specific pathologic issues: left ventricular dilation, wall hypertrophy (concentric or asymmetric), reduction of function (visual ejection fraction), segmental wall motion abnormality (yes or no), right ventricular dilation, left atrial dilatation, aortic root dilatation, valve calcification, pericardial effusion, pleural effusion, and dilation with reduced inspiratory reactivity of inferior vena cava.

## 10 CONCLUSION

Performing POCUS is an accurate, affordable, accessible, and comprehensive tool. It has a fast learning curve, and can prevent unnecessary and more expensive imaging.^6^, ^9^ POCUS can serve as a screening tool and guide management of patients with valvular heart disease.^54^ Thus, it is important to acknowledge the limited training availability, lack of simplified screening protocols, and importantly reimbursement. As the utilization of POCUS increases in the outpatient clinic, more research is needed about its impact on screening, management, outcomes and cost of care.

## Data Availability

No new data generated
